# Assessing Cannabis Use in People with Psychosis

**DOI:** 10.1089/can.2023.0032

**Published:** 2024-02-12

**Authors:** Edward Chesney, Will Lawn, Philip McGuire

**Affiliations:** ^1^ Department of Psychosis Studies, Institute of Psychiatry, Psychology and Neuroscience, King's College London, London, United Kingdom.; ^2^ Department of Psychology, Institute of Psychiatry, Psychology and Neuroscience, King's College London, United Kingdom.; ^3^ Department of Psychiatry, Oxford University, Warneford Hospital, Oxford, United Kingdom.; ^4^ NIHR Oxford Health Biomedical Research Centre, Oxford, United Kingdom.

**Keywords:** cannabis, cannabis use disorder, psychosis, schizophrenia, assessment, urinalysis, toxicology

## Abstract

**Introduction:**

Cannabis use is common in people with psychotic disorders and is associated with the exacerbation of symptoms, poor treatment adherence, and an increased risk of relapse. Accurate assessment of cannabis use is thus critical to the clinical management of psychosis.

**Discussion:**

Cannabis use is usually assessed with self-report questionnaires that were originally developed for healthy individuals or people with a cannabis use disorder. Compared to these groups, the pattern of cannabis use and the associated harms in patients with psychosis are quite different. Moreover, in people with psychosis, the accuracy of self-reported use may be impaired by psychotic symptoms, cognitive deficits, and a desire to conceal use when clinicians have advised against it. Although urinary screening for delta-9-tetrahydrocannabinol is sometimes used in the assessment of acute psychotic episodes, it is not used in routinely. Cannabis use could be assessed by measuring the concentration of cannabinoids in urine and blood, but this is rarely done in either clinical settings or research.

**Conclusion:**

Using quantitative biological measures could provide a more accurate guide to the effects of use on the disorder than asking patients or using questionnaires.

## Introduction

The prevalence of cannabis use in patients with psychosis is very high,^1,2^ with as many as 36% of patients with first-episode psychosis and 21% of those with established schizophrenia meeting diagnostic criteria for a cannabis use disorder.^3^ Moreover, in people with psychosis, cannabis use can have a major effect on the course of the disorder: it is associated with more severe symptoms, an increased risk of relapse and violence, longer hospital admissions, and a lower quality of life.^4–7^ These effects appear to be dose-dependent, with worse outcomes in frequent users and users of high-potency strains.^8,9^ A recent study from Denmark found that almost half of the harm associated with cannabis use across the entire population was observed in patients with schizophrenia.^10^

The harms associated with cannabis in psychosis populations appear to be increasing. Between 2000 and 2016, the incidence of “cannabis-induced psychosis” increased by 67% in Norway, 115% in Denmark, and 238% in Sweden.^11^ In Canada, the number of patients presenting to emergency departments with a “cannabis-induced psychoses” doubled between 2015 and 2019.^12^ This is a major issue, as many of these individuals subsequently develop a psychotic disorder.^13^ In a survey conducted in the United States, the proportion of people with a self-reported diagnosis of a psychotic disorder who also reported daily cannabis use increased from 3% in 2001 to 8% in 2012.^14^ These trends may be explained by softening societal attitudes to cannabis use, alongside decriminalization and legalization in several jurisdictions.^15–17^ Another factor may be an increase in the potency of illicit cannabis: since the 1990s, the average concentration of delta-9-tetrahydrocannabinol (THC) quadrupled in the United States, from 4% to 15%, and doubled in Europe, from 6% to 11%.^18^

Patients with psychosis who stop using cannabis have better outcomes than those who continue to use the drug.^5^ However, at present, there are no evidence-based pharmacological or psychological treatments to reduce or stop cannabis use,^19^ an important unmet clinical need. Progress in developing new interventions may have been hampered by the lack of standardized assessments for cannabis use.^20^ Clinical guidelines for assessing drug use are vague, simply suggesting that clinicians should assess patterns of drug use and that biological tests “may be useful.”^21^

## Quantifying Cannabis Exposure via Self-Report

Several aspects of cannabis use can be assessed: frequency of use, total amount of cannabis used, time spent intoxicated, the subjective effects of intoxication, withdrawal symptoms, motivation to use, desire to quit, functional impairment, and the presence of cannabis use disorder or dependence. A summary of some of the most established self-rating scales is provided in Table 1. From a clinical perspective, assessing total cannabis exposure is important, as it has a dose–response relationship with key clinical outcomes.^8,9^

**Table 1.** Selected Scales for Structured Assessment of Cannabis Use, Cannabis Use Disorder, Cannabis Withdrawal, and Other Harms

**Measure**	**Aim**	**Items**	**Example questions**	**Validation/psychometric assessment in a psychosis population**
Timeline Followback Method (TLFB)^23^	Quantify recent exposure	From 7 days to 3 months	Can you remember what you did last Saturday? How many joints did you smoke that day?	Hjorthøj et al.^24^
Daily Sessions, Frequency, Age of Onset, and Quantity of Cannabis Use Inventory (DFAQ-CU)^25^	Quantify recent exposure and age of onset	41	On a typical day you use marijuana, how many sessions do you have? How many times a day, on a typical weekend, do you use cannabis?	No
Severity of Dependence Scale (SDS)^26^	Assess severity of dependence	5	Did you think your use of cannabis was out of control? How difficult did you find it to stop, or go without cannabis?	Hides et al.^27^
Cannabis Abuse Screening Test (CAST)^28^	Screen for cannabis use disorders	6	Have you smoked cannabis before midday? Have friends or members of your family told you that you ought to reduce your cannabis use?	No
Cannabis Use Disorders Identification Test–Revised (CUDIT-R)^29^	Screen for cannabis use disorders	8	How often do you use cannabis? How often during the past 6 months did you fail to do what was normally expected from you because of cannabis?	No
The Alcohol, Smoking and Substance Involvement Screening Test (ASSIST)^30^	Screen for substance use disorders	7	In the past three months, how often have you… … used cannabis? … had a strong desire or urge to use cannabis? … failed to do what was normally expected of you because of your use of cannabis?	Hides et al.^31^
Marijuana Withdrawal Checklist (MWC)^32^	Assess withdrawal symptoms	22	Indicate how much you are feeling each symptom right now: “Irritability,” “Craving,” “Headaches,” “Sleep problems”	No
Cannabis Withdrawal Scale (CWS)^33^	Assess withdrawal symptoms	19	I had some angry outbursts Nightmares or strange dreams Trouble getting to sleep	No
Cannabis Experiences Questionnaire (CEQ)^34^	Assess symptoms of intoxication	42	How often do you have these experiences when smoking cannabis? “Enhanced perceptual awareness,” “Ecstatic,” “Paranoid,” “Depressed”	Birnbaum et al.^35^
		13 (Short version)		
Marijuana Craving Questionnaire (MCQ)^36^	Assess craving	47	I would do almost anything for a joint Smoking marijuana would help me sleep at night	No
		12 (Short version)		
Obsessive Compulsive Drug Use Scale for Cannabis (OCDUS-CAN)^37^	Assess craving	12	If you don't use, how often do you feel the urge or drive to use cannabis? How much control do you have over your cannabis use?	Dekker et al.^38^

**FIG. 1.**
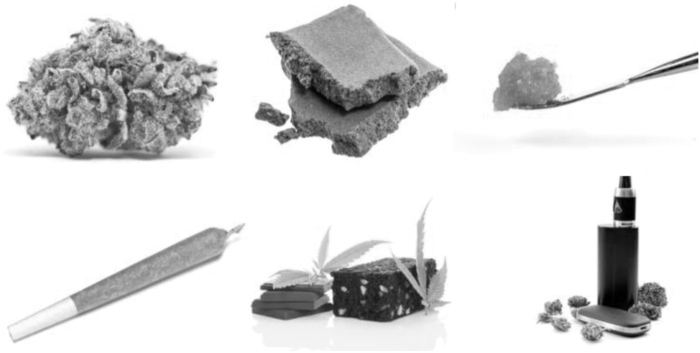
The increased diversity of cannabis formulations and methods of administration has complicated the assessment of cannabis exposure.

Accurately quantifying cannabis use is difficult as there is no standardized unit of cannabis.^22^ Users of cannabis may differ in their frequency of use, the number of joints they use each day, joint size or amount per session, formulation (flower, resin, edible, concentrate), potency (i.e., % concentration of THC), and method of administration (smoked, vaporized, or oral) (Fig. 1).

Perhaps the single most important variable is the frequency of use, normally recorded in days of use per week or month. Frequency is relatively easy to measure, reliable, and has strong associations with cannabis dependence.^39^ Its main limitation as a metric is that it does not differentiate between a user who smokes a single small joint each evening and another who uses several grams of cannabis throughout the day. This is particularly important for populations with psychosis, as daily use is relatively common. In a recent European study, 45% of all patients with psychosis who had ever used cannabis reported that they were or had been daily smokers, compared to just 15% of controls.^1^ Estimates of joint size and potency can be inaccurate, an issue which has likely worsened with the arrival of novel formulations such as cannabis concentrates (Fig. 1).^40,41^ Other aspects, such as route of administration and sharing, make estimating total cannabis exposure even more complicated.^42^ Even if a self-report assessment was able to measure the exact amount of cannabis used, it would still not account for the large intra- and inter-subject variation in bioavailability. When cannabis is smoked, for example, these estimates range from 2% to 56%.^42^

The Gold-standard *method* for collecting self-reported cannabis use data is the Timeline Followback (TLFB).^23,43^ To complete an assessment, the participant records whether or not they used cannabis on each day over the past week, month, or longer. Most studies use the TLFB to record the number of joints per day, although additional information regarding joint size, formulation, potency, and method of administration can also be collected.^44^ However, the TLFB is yet to be comprehensively empirically tested as an assessment for cannabis exposure. Its incremental validity, the extent to which a measure provides unique information when used alongside existing tests, should be further examined. In one study, the average grams per cannabis administration, assessed using the TLFB method, had stronger associations with urine cannabinoid levels and cannabis-related harms than simply assessing frequency or quantity of use.^45^ However, in other studies results have been less encouraging.^46,47^ A further limitation of the TLFB is that it is time-consuming, complex, and its accuracy depends on the expertise of the assessor and engagement of the user. In research studies, there may be time to complete thorough assessments, but in clinical settings professionals will rarely have the time to collect such detailed data. The majority of clinical trials in populations of patients with psychosis and comorbid cannabis use disorder have used the TLFB method to assess frequency of use and/or quantity of cannabis consumed (Table 2). However, major clinical trials and epidemiological studies which recruit general psychosis patients have used a much more heterogenous range of assessments (Table 3).

**Table 2.** Cannabis-Related Outcome Measures Used in Clinical Trials of Comorbid Psychosis and Cannabis Use Disorder

**Study**	**Population**	**Intervention**	**Self-report measures of cannabis exposure**	**Scales**	**Biochemical measures**
CapOpus^48^	Psychosis and cannabis use disorder, age 18–35, and managed by an early intervention team (*n*=103)	Motivational interviewing and cognitive behavioral therapy vs. treatment as usual	Number of days cannabis use in the past month, assessed using TLFB “Standard” joints per month. Defined as 0.17 g high-potency cannabis or 0.5 g of cannabis resin, assessed using TLFB	None	Plasma THC, THC-OH, and THC-COOH concentration
Rabin et al.^49^	Schizophrenia or schizoaffective disorder and cannabis dependence (*n*=19)	Single-arm trial of contingency management and individual supportive therapy	Cannabis in grams/day over 4 weeks, assessed using TLFB	Marijuana Withdrawal Checklist	Qualitative urinalysis Creatinine-normalized urine THC-COOH concentration
CIRCLE^50^	Psychosis and cannabis use disorder, age 18–36, and managed by an early intervention team (*n*=551)	Contingency management (up to £240)+psychoeducation vs. psychoeducation alone	Number of days cannabis use in the past 3 months, assessed using TLFB	None	Qualitative urinalysis
Smeerdijk et al.^51^	Recent-onset schizophrenia (*n*=75) and their parents	Family motivational intervention vs. routine family support	Number of days cannabis use in the past 3 months, assessed using TLFB Cannabis in grams/day assessed using TLFB	Obsessive Compulsive Drug Use Scale	Qualitative urinalysis
Schnell et al.^52^	Schizophrenia or schizoaffective disorder and cannabis abuse or dependence (*n*=30)	Clozapine vs. ziprasidone	Joints per month, assessed using a detailed interview	Stages of Change Readiness and Treatment Eagerness Scale	Qualitative urinalysis and toxicological hair analysis

THC, delta-9-tetrahydrocannabinol; THC-COOH, 11-nor-9-carboxy-THC; THC-OH, 11-hydroxy-THC; TLFB, Timeline Followback.

**Table 3.** Cannabis Exposure-Related Outcome Measures Reported in Major Clinical Trials and Epidemiological Studies in Psychosis Populations

**Study**	**Design**	**Population**	**Self-report measures of cannabis exposure**	**Other Assessments**	**Biochemical measures**
CATIE^53^	Clinical trial of antipsychotic medications	Schizophrenia (*n*=1493)	Current use (Y/N)	None	Qualitative urinalysis Hair radioimmunoassay
EUFEST^54^	Clinical trial of antipsychotic medications	First-episode schizophrenia (*n*=323)	Frequency of use (days/month)	DSM-IV criteria	None
OPTIMISE^55^	Clinical trial of antipsychotic medications	Schizophrenia and schizophreniform disorder (*n*=446)	Frequency of use, amount used, and route of administration	DSM-IV criteria	None
PAFIP^56^	Clinical trial of antipsychotic medications	First-episode psychosis (*n*=376)	Baseline: current vs. non-users Follow-up: persistent users, ex-users, and never-users	Excluded if met DSM-IV criteria for drug dependence	None
AESOP^57^	Case–control study	First-episode psychosis (*n*=511) and controls (*n*=412)	Ever use (verified using case-notes review and collateral history)	None	None
EUGEI^58,59^	Case–control study	First-episode psychosis (*n*=901), clinical high risk (*n*=316), and controls (*n*=1237)	Cannabis Experiences Questionnaire (includes questions on frequency of use, cannabis potency, and amount per use)	None	Quantitative analysis of plasma
NAPLS-2^60^	Case–control study	Clinical high risk (*n*=764) and controls (*n*=280)	Current use (Y/N) Lifetime use (Y/N) Frequency of use	DSM-IV criteria	None

DSM-IV, Diagnostic and Statistical Manual of Mental Disorders, fourth edition.

## Additional Limitations of Self-Report Measures in Psychosis Populations

The reliability and validity of self-report measures depend on the individual collecting the data, the individual being assessed, and the rationale for and context of testing. A fundamental issue with studies comparing self-reported drug use with an objective test is that the expectation of testing itself may increase the likelihood of honest disclosure and therefore artificially enhance the supposed validity of the self-report measure. Despite this, a significant proportion of people are still hesitant to disclose their illicit drug use in such studies. In a large sample of healthy young people from the United States, only 61% of those with a THC positive urine sample reported that they had used cannabis in the past month.^61^ Perhaps the only exception to this rule are patients who volunteer for treatment programs or clinical trials for substance use disorders, as they recognize that their drug use is causing harm and are seeking support to reduce it.^62^ This is a major issue, as most of the data supporting the validity self-reported cannabis use are from studies which recruited this type of patient.^63^ The effect of this issue on the reliability of self-report measures is demonstrated by comparing two clinical trials in patients with psychosis: CapOpus and CATIE.

CapOpus randomized 103 patients with psychosis who used cannabis to either motivational interviewing and cognitive behavioral therapy or treatment as usual with the aim of reducing their cannabis use.^24^ It found moderately strong correlations between self-reported number of days of cannabis use (*r*=0.49) and number of joints smoked per month (*r*=0.49) with the plasma concentration of THC, which increased after exclusion of extreme outliers (*r*=0.75 and *r*=0.83, respectively). The CATIE trial recruited patients with psychosis, with or without current cannabis use, and randomized them to different oral antipsychotics. The study was designed to compare their effectiveness at treating psychotic symptoms, not to reduce problematic substance use.^53^ Of the 168 participants who had a positive urine or hair test for cannabis in CATIE, almost half (38%) denied that they had used cannabis in the past 90 days. Thus, even when participants knew that they were going to be tested, self-report was unreliable in patients who hadn't actively volunteered to reduce their cannabis use.

In the CapOpus trial, correlations between self-report and plasma THC levels were weaker as symptom severity increased (either total or negative symptoms), but impaired cognition (as measured by a verbal learning task) did not impact the correlation between self-reported cannabis use and plasma THC levels. In the CATIE trial, older age, non-White race, and criminal proceedings were all associated with under-reporting, as were positive psychotic symptoms and impaired cognition.^53^

Further evidence demonstrating the unreliability of self-report in people with psychosis comes from a study of 203 patients with schizophrenia.^64^ Just 33 (16%) participants reported illicit substance use within the past 3 months despite 67 (33%) returning a positive hair or urine sample. In another study of forensic patients, under close supervision and with requirements to abstain from drug use, the accuracy of self-report was even worse.^65^ Of 37 patients with a positive urine drug screen, the majority (70%) denied recent drug use. Tampering of samples was also an issue: 10% of samples were suspiciously dilute and three patients returned consecutive samples with remarkably similar creatinine levels. Together these studies demonstrate how self-report measures are unreliable in psychosis populations. Despite this, few studies in psychosis populations report objective measures of cannabis use (Table 3).

## Quantitative Biochemical Assessment of Cannabis Exposure

**FIG. 2.**
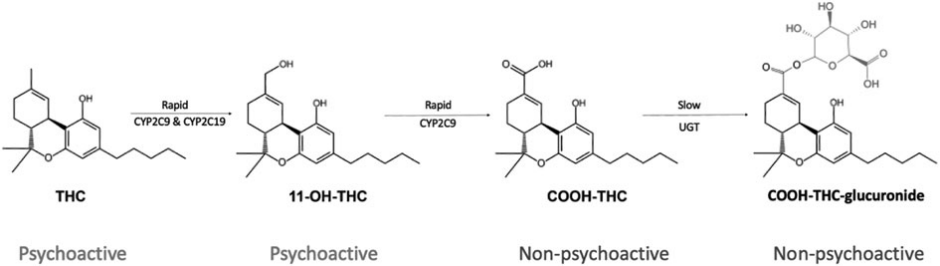
Simplified diagram of THC metabolism. THC, delta-9-tetrahydrocannabinol.

The limitations of self-reported cannabis use in psychosis populations suggest that objective analyses of biological samples may be necessary to obtain an accurate assessment. In this study, we consider which analyses provide the best assessment of overall cannabis exposure, as well as recent use. The main psychoactive constituent of cannabis is THC which is rapidly metabolized to an active metabolite 11-hydroxy-THC (Fig. 2). The concentration of THC is highest during smoking, while 11-hydroxy-THC concentration peaks soon after. 11-hydroxy-THC is converted to carboxy-THC, which is nonpsychoactive and is the most prevalent metabolite in plasma. THC and its metabolites can be measured in blood, urine, saliva, hair, breath, and sweat (Table 4).^43,66,67^

**Table 4.** The Advantages and Disadvantages of Testing Different Biological Matrices for THC and Its Metabolites in Cannabis Smokers

**Biological Matrix**	**Acceptability**	**Standard analytes**	**Maximum detection window**	**Advantages**	**Limitations**
Urine	High	Carboxy-THC	Infrequent users: several days Heavy users: weeks–months	Immunoassays can provide an immediate qualitative result Quantitative measurement of carboxy-THC will provide a reasonable estimate of total cannabis exposure if creatinine corrected	Not a reliable biomarker for recent use in frequent users
Blood	Medium	THC Hydroxy-THC Carboxy-THC	THC and hydroxy-THC: several hours Carboxy-THC and carboxy-THC-glucoronide: several weeks	THC and hydroxy-THC can be used as biomarkers of recent use in occasional users Quantitative measurement of carboxy-THC will provide a reasonable estimate of total cannabis exposure	Not a reliable biomarker for recent use in frequent users
Hair	Low	Carboxy-THC	Months	A long detection window (up to 3 months) means that it can be used to identify historic use	It is questionable whether quantitative measures are valid biomarkers as exposure to environmental factors such as sunlight can affect results considerably
Saliva	High	THC	Several hours	Immunoassays can be used to exclude recent use	Short detection window. Results may be affected by recent consumption of food or drinks.
Sweat	Low	THC	1 week	Cumulative exposure can be measured for up to 7 days.	Dose-response relationship is unreliable
Breath	High	THC	Several hours	May be a useful biomarker for identifying recent use (i.e., <4 h)	Not established or validated

Cannabinoids are highly lipophilic; they build up in fatty tissues and as a result have a very long terminal elimination half-life (>5 days).^42^ As a result, many heavy cannabis users will have positive plasma and urine samples even after a month of abstinence, and simple immunoassay tests cannot be used to confirm recent abstinence.^68,69^ Furthermore, for many patients, abstinence may be an unreasonably ambitious objective and a harm reduction approach may be more realistic. In this group, immunoassay tests are also not useful as they do not provide a quantitative result which could demonstrate changes in the amount of cannabis use over time.

The biomarker used in most clinical trials is creatinine-corrected urine carboxy-THC. Carboxy-THC has an initial urinary excretion half-life of about 1.4 days (range=1.0–2.3) in frequent smokers, making it a reasonable biomarker for cannabis exposure.^70^ To our knowledge, only one study has measured creatinine-corrected carboxy-THC in patients with psychosis (Table 2). Rabin et al. performed gas chromatography-mass spectrometry on the urine of 13 cannabis-dependent patients with schizophrenia and 13 controls with cannabis dependence and no other psychiatric comorbidities.^49^ They found that the creatinine corrected-carboxy-THC (THC-COOH) was 431±421 ng/mg in patients compared to 882±917 ng/mg in controls (*p*=0.12), in keeping with the amount of cannabis that each group reported that they used (1.22 ± 0.8 grams per day vs. 1.63 ± 1.2 grams per day [*p*=0.21], respectively). In another recent study, Barguil et al. collected hair samples from four groups of patients: acute cannabis-induced psychosis, schizophrenia and other chronic psychoses, personality and mood disorders, and a control group of cannabis users hospitalized for a nonpsychiatric illness.^71^ Perhaps counterintuitively, the lowest mean THC concentration was found in the acute cannabis-induced psychosis group, 0.16 ng/mg (95% confidence interval [CI]=0.016–0.30). The schizophrenia group had a concentration eight times higher, 1.3 ng/mg (95% CI=0.78–1.73). The personality and mood disorder group and the control group had concentrations in between: 0.29 ng/mg (95% CI=0.16–0.43) and 0.44 ng/mg (95% CI=0.23–0.65), respectively. It is unclear whether these observations are solely due to differences in the total cannabis exposure between the groups or may also be due to differences in exposure to environmental factors that can reduce cannabinoid concentrations, such as sunlight and the use of cosmetic hair treatments^72,73^: patients with severe mental illness may be less frequently exposed to these factors.^74^

## The Potential Advantages of Obtaining Quantitative Biological Assessment of Cannabis Exposure in Psychosis Populations

Quantitative biochemical measurement of cannabinoids could be used to track treatment progress. While the data from people with psychosis are limited, their potential has been demonstrated in several clinical trials of cannabis use disorder.^75–77^ Each of these trials demonstrated significant differences between treatment groups using urine carboxy-THC as an outcome, despite relatively small sample sizes. Further research is needed to establish whether collecting serial urine samples is valuable at the individual level, particularly as it may not be as informative in users with inconsistent or binge patterns of use. The choice of biomarker will depend on the nature of the treatment program, clinical trial, or epidemiological study in question, and it is important to carefully consider the metabolite (or metabolites) to analyze, as well as which biological medium to sample (Table 4) in each case.

Offering quantitative tests to patients may also promote therapeutic alliance and engagement with mental health treatment.^78^ It may demonstrate that mental health services are in tune with patients' needs and interests. Many cannabis users are interested in cannabis science and are aware of the range of cannabinoids found in cannabis. This might provide a rationale for measuring a broader profile of compounds, such as cannabidiol, delta-8-THC, and terpenes. However, whether demonstrating to a heavy user that the concentration of cannabis in their body is several times over a limit for safe use would encourage them to moderate their cannabis consumption remains unclear.^79^

Standardized objective assessments will enable accurate comparisons between research studies across populations and time. Other benefits include the simplicity of data collection, particularly for clinical services who can collect urine and plasma samples without having to train staff to use complex and time-consuming self-report measures. Recently, novel point of care technologies have been developed to measure medication levels using finger-prick samples of blood, a method which could also be used to test for concentrations of cannabinoids.^80^ Quantitative assessments will also address tampering of urine samples as the samples are creatinine-corrected, a feature which may be particularly useful in high-risk settings, such as forensic services. The main limitation of quantitative analysis is the cost, but in comparison to the costs associated with other medical investigations, hospital admission, or even the cost of a clinician's time to complete an in-depth assessment of substance use, these are small.

## Conclusions

There is no gold-standard assessment for assessing cannabis use in people with psychosis. Self-report methods are not accurate, partly because patients are dis-incentivized to disclose their drug use, and because the psychotic and cognitive symptoms that are part of the disorder can impair accurate recall. Quantifying cannabis exposure by measuring cannabinoids in biological samples may prove to be particularly useful both clinically and in research (Table 5). Studies should establish whether creatinine-corrected urine THC-COOH concentration could serve as the gold-standard objective measure of cannabis exposure. Its validity should be further scrutinized in both healthy and psychosis populations, particularly in terms of temporal reliability in consistent users. Further investigation of its value in aiding psychiatric diagnosis and formulation, determining thresholds for risky use, monitoring treatment progress in individual patients, and promoting engagement and therapeutic alliance is worthwhile.

**Table 5.** Suggested Approaches to the Assessment of Cannabis Exposure According to Population and Setting

**Setting**	**Population**	**Recommendation**
Clinical	Psychosis, engaged with treatment	Further research is required to investigate the validity of different self-report measures, particularly for patients with severe cognitive or psychosis symptoms Qualitative immunoassays may aid honest disclosure of recent drug use
Clinical	Psychosis, not engaged with treatment	Self-report measures should be interpreted with caution Quantitative biochemical tests
Clinical	Psychosis, high-risk/forensic	Qualitative immunoassay tests may be subject to tampering; quantitative biochemical tests (plasma>urine) are indicated
RCT	Psychosis with cannabis use disorder Cannabis use as a primary outcome	In depth self-report measures such as TLFB or ecological momentary assessment may be worthwhile Quantitative biochemical tests
RCT	Psychosis with cannabis use disorder Cannabis use as a secondary outcome	Concise self-report measures may be sufficient Quantitative biochemical tests
Epidemiological study	General population	Concise self-report measures alongside quantitative biochemical tests
Epidemiological study	Psychosis populations	Concise self-report measures alongside quantitative biochemical tests

RCT, randomised controlled trial.

## Abbreviations Used

CI: confidence interval
THC: delta-9-tetrahydrocannabinol
THC-COOH: 11-nor-9-carboxy-THC
THC-OH: 11-hydroxy-THC
TLFB: Timeline Followback
